# Genome-Wide Identification and Expression Analysis of the Copper Transporter (*COPT*/*Ctr*) Gene Family in *Kandelia obovata*, a Typical Mangrove Plant

**DOI:** 10.3390/ijms242115579

**Published:** 2023-10-25

**Authors:** Quaid Hussain, Ting Ye, Sihui Li, Jackson Nkoh Nkoh, Qiao Zhou, Chenjing Shang

**Affiliations:** 1 Shenzhen Key Laboratory of Marine Bioresource and Eco-Environmental Science, College of Life Sciences and Oceanography, Shenzhen University, Shenzhen 518060, China; quaid_hussain@yahoo.com (Q.H.); 2100252001@email.szu.edu.cn (T.Y.); 18138206919@163.com (S.L.); nkohjackson@szu.edu.cn (J.N.N.); zhouqiao@szu.edu.cn (Q.Z.); 2 College of Physics and Optoelectronic Engineering, Shenzhen University, Shenzhen 518060, China

**Keywords:** copper stress, copper transporter, *Kandelia obovata*, qRT-PCR, 3D structures

## Abstract

The copper transporter (*COPT*/*Ctr*) gene family plays a critical part in maintaining the balance of the metal, and many diverse species depend on *COPT* to move copper (Cu) across the cell membrane. In *Arabidopsis thaliana*, *Oryza sativa*, *Medicago sativa*, *Zea mays*, *Populus trichocarpa*, *Vitis vinifera*, and *Solanum lycopersicum,* a genome-wide study of the COPT protein family was performed. To understand the major roles of the *COPT* gene family in *Kandelia obovata* (*Ko*), a genome-wide study identified four *COPT* genes in the *Kandelia obovata* genome for the first time. The domain and 3D structural variation, phylogenetic tree, chromosomal distributions, gene structure, motif analysis, subcellular localization, cis-regulatory elements, synteny and duplication analysis, and expression profiles in leaves and Cu were all investigated in this research. Structural and sequence investigations show that most *KoCOPTs* have three transmembrane domains (TMDs). According to phylogenetic research, these *KoCOPTs* might be divided into two subgroups, just like *Populus trichocarpa*. *KoCOPT* gene segmental duplications and positive selection pressure were discovered by universal analysis. According to gene structure and motif analysis, most *KoCOPT* genes showed consistent exon–intron and motif organization within the same group. In addition, we found five hormones and four stress- and seven light-responsive cis-elements in the *KoCOPTs* promoters. The expression studies revealed that all four genes changed their expression levels in response to copper (CuCl_2_) treatments. In summary, our study offers a thorough overview of the *Kandelia obovata COPT* gene family’s expression pattern and functional diversity, making it easier to characterize each *KoCOPT* gene’s function in the future.

## 1. Introduction

Copper (Cu) is a crucial micronutrient for all living things and a protein cofactor essential for many physiological activities [1]. However, high concentrations of Cu could cause pollution that slows plant growth and development [2]. According to Cano-Gauci and Sarkar [3], Cu reactivity can cause significant cell oxidative damage, reduced root growth, flowering problems, and germination defects. In response to Cu shortages and excesses, eukaryotes have developed a system to regulate the acquisition and distribution of Cu precisely. Cu is linked to several physiological processes in plants, including ethylene sensing, cell wall metabolism, superoxide scavenging, mitochondrial respiration, and photosynthesis [4], and its deficiency results in numerous abnormal phenotypes in plants, including reduced water transport, distorted young leaves, and reduced growth and reproductive development [5].

One of these techniques is expressing the *COPT*/*Ctr* family of membrane proteins, which are conserved and function as Cu transporters [6]. There have been reports of several transporter protein varieties that can mediate Cu absorption. The COPT (COPper Transporter)/Ctr (copper transporter) proteins, which are members of numerous protein families in various organisms, make up the largest group [7]. A *Ctr-like* Cu transporter family with a high degree of conservation includes the *COPT*/*Ctr* family. Three TMDs—TMDs 1, 2, and 3—are found in most *COPT*/*Ctr* transporters. Most *COPT*/*Ctr* transporters include methionine motifs in their N termini that are critical for Cu binding and enhanced import [8]. The MxxxM motif, critical for controlling transporter pore size and necessary for Cu transporter function, is found in TMD2 [4,9]. The GxxxG motif is present in TMD3, which is important for the close helix packing of the three TMDs and for forming a structurally and functionally mature transporter [4,9].

The first *COPT*/*Ctr* transporter was identified as a plasma membrane protein in *S. cerevisiae* (*ScCTR1*) necessary for high-affinity Cu transfer into the cell [10]. The *COPT*/*Ctr* family was discovered in plants by functional yeast complementation or sequence homology to other eukaryotic *Ctr* Cu transporters [11]. Six *AtCOPT* genes are found in *Arabidopsis thaliana* [12]. When Cu is limited, *AtCOPT1* contributes to soil Cu absorption [13]. According to Perea-Garca et al. [14], *AtCOPT2* plays a role in acquiring Cu and cross-talk across iron-deficient responses. According to Klaumann et al. [15], *AtCOPT5* is crucial for Cu export from the vacuole and involves transporting Cu from the root to reproductive organs.

The *AtCOPT6* gene is expressed in response to both Cu excess and deficiency, following [16]. Seven members comprise the rice *COPT*/*Ctr* family. According to [1], *OsCOPT2*, *OsCOPT3*, and *OsCOPT4* may collaborate with *OsCOPT6*, whereas *OsCOPT7* functions independently for Cu transport. Interestingly, iron (Fe), zinc (Zn), or manganese (Mn) concentrations affected these genes’ expression [1]. Eight putative Cu transporter genes (*VvCtrs*) are present in the grape (*Vitis vinifera* L.) genome. *CuSO4* availability uniquely impacted *VvCtr1* and *VvCtr8*, and Zn stimulation and salt stress suppressed *VvCtr8* expression, respectively [17]. According to these findings, most *COPT*/*Ctr* families participate in Cu uptake, transport, and distribution and are susceptible to other metal influences. Although the *COPT*/*Ctr* family members have been extensively researched in herbaceous plants, very few investigations on woody plants, including *Populus trichocarpa* [6] *and Vitis vinifera* [18], have been reported.

Mangroves are the predominant halophytic vegetation and have substantial ecological significance in tropical and subtropical coastal wetlands. They are also well adapted to these highly challenging intertidal environments [19,20]. A woody plant called *Kandelia obovata* is mainly found in salt marshes in tropical and subtropical regions from East Asia to Southeast Asia [21,22,23]. *Kandelia obovata* overcomes cyclic and aperiodic tidal influences, which result in high salinity, severe erosion, and anaerobic conditions [24], to adapt to transitional environments where the land and ocean meet. In order to preserve biodiversity and stop erosion, *Kandelia obovata* is essential [25,26]. In China, the widely cultivated and frequently utilized plant species known as *Kandelia obovata* vary depending on the age of the mangrove forest. Since the 1960s, multiple mangrove plantations, primarily of the species *Kandelia obovata*, have been successfully carried out to protect the sea bank in this area. As a result, *Kandelia obovata* mangrove forests of various ages have been planted [27]. According to Wang et al. [28], *Kandelia obovata* mangroves are more resilient to waterlogging and respond to different light irradiances. Cu, lead (Pb), and zinc (Zn) are the most prevalent heavy metal pollutants, and mangroves serve as a significant “pool” of these metals [29,30,31]. Seedlings of *Kandelia obovata* grew less when exposed to Cu [32,33,34]. The simultaneous exposure to several heavy metal stresses might result in a significant degradation of chlorophyll in the leaves of *Kandelia obovata* [35]. Shen et al. [34] observed a decrease in the overall chlorophyll content of *Kandelia obovata* when it was subjected to synthetic wastewater containing a combination of heavy metals. According to Zhao and Zheng [33], when the concentration of Cu increased, the amount of soluble sugar in the roots of *Kandelia obovata* seedlings grew first and then declined, whereas the amount of soluble sugar in the leaves decreased [34]. However, the effect of Cu-induced stress on the *COPT*/*Ctr* transporters has not been given enough attention since most studies on this species focused on physiological and molecular response.

Basically, the study by Shen et al. [34] observed that this species showed different tolerance levels for different heavy metals, and it could tolerate up to 400 mg/L of Cu stress; hence, we considered concentrations varying from 0 to 400 mg/L of Cu solution. Thus, this study performed a genome-wide analysis of the *Kandelia obovata COPT*/*Ctr* transporters under different Cu stress levels. The analysis included physicochemical characteristics, chromosomal locations, phylogenetic trees, gene structures, motif investigations, cis-regulatory elements, synteny and duplication analysis, and expression profiles. Our findings lighten *KoCOPTs*’ functionality by profiling expression and utilizing the promoter architecture. We also investigated the cis-regulatory elements’ role in the *COPT*/*Ctr* transporters’ promoter sequences to examine how they influence hormones in various physiological and biological processes. Furthermore, under Cu treatment circumstances, the expression profiles of *KoCOPTs* in the leaves have been examined. Future research into the functions of these potential *COPT*/*Ctr* genes in *Kandelia obovata* under metal stressors may benefit from our findings.

## 2. Results 

### 2.1. Copper Treatments Affect the Growth of Kandelia obovata Seedlings 

**Figure 1:**
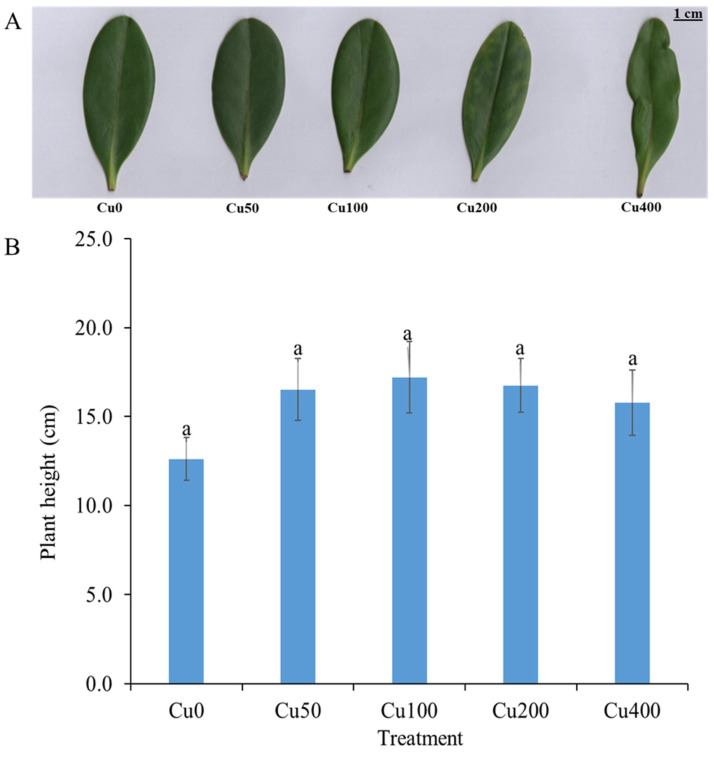
The growth and leaf structure of *Kandelia obovata* seedlings. The provided photos in (**A**) depict representative leaf structures, while (**B**) illustrates the growth of plants subjected to varying copper treatments, specifically Cu0, Cu50, Cu100, Cu200, and Cu400 mg/L. The vertical axis shows the plant height in centimeters and the horizontal axis shows different treatments of Cu. The same letters on the horizontal bar indicate that there was no significant difference in plant height at the 5% level.

Figure 1A displays images showing the distinct variations in leaf structure among the five treatments (Cu0, Cu50, Cu100, Cu200, and Cu400 mg/L). A noticeable reduction in leaf thickness was observed in the Cu400 treatment, whereas an increase in leaf width was observed in the Cu50 and Cu100 treatments compared to the Cu0 treatment (Figure 1A). In contrast to the other copper (Cu) treatments, it was shown that Cu400 led to a significant increase in leaf length. The plant height of *Kandelia obovata* did not show any notable variations among the four treatments (Cu50, Cu100, Cu200, and Cu400 mg/L) and the control without heavy metals (Cu0) (Figure 1B). The plant height of seedlings subjected to Cu stress at concentrations of Cu50, Cu100, Cu200, and Cu400 (CuCl_2_) grew by 31%, 37%, 33%, and 25%, respectively, compared to the control group (Cu0) (Figure 1B).

### 2.2. Identification of COPT Family Members in Kandelia obovata

**Figure 2:**
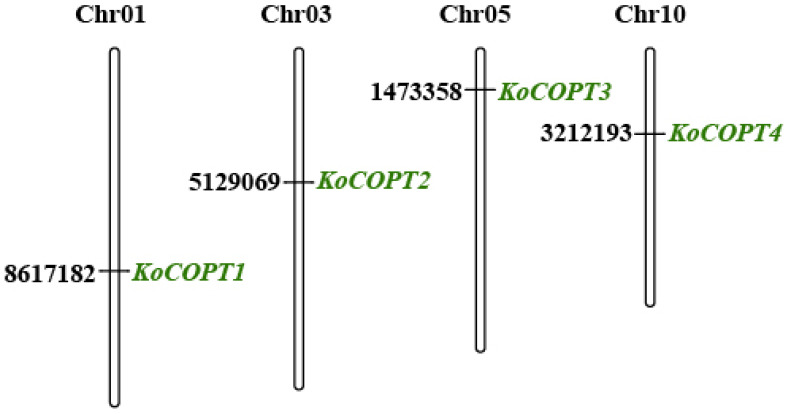
*COPT* gene distribution on *Kandelia obovata*’s four chromosomes is shown schematically, along with the gene’s name in green on the right side. The black lettering on the chromosomes indicates the location of the *COPT* genes. The top of each chromosome (Chr) is where you may find the chromosomal numbers.

*Kandelia obovata*’s genome contains four *COPT* genes in total, which is a lot more than the previously reported *COPTs* in other plant species like *Arabidopsis thaliana* (At), *Oryza sativa* (Os), *Medicago sativa* (Ms), *Zea mays* (Zm), *Populus trichocarpa* (Pt), and *Vitis vinifera* (Vv). The molecular weight of the *COPT* family ranged from 11.18 to 17.09, with an average of 14.67 kDa. *KoCOPT1* had the highest pI, 9.72, while *KoCOPT3* had the lowest pI, 6.82. The *COPT* family’s average isoelectric point (pI) ranged from 6.82 to 9.72. Grand average hydropathy index (GRAVY) values of four *COPTs* ranged from 0.309 to 0.805, indicating a hydrophobic character. Understanding the molecular function will be easier by identifying the subcellular distribution of COPT proteins. According to the subcellular localization prediction of COPT proteins, four COPTs were likely found in the plasma membrane (Table 1). The *COPT* family had an average of 106 to 159 amino acids, an aliphatic index of 82.2 to 124.66, and an instability range of 34.84 to 49.66. The MapChart web service used the chromosomal positions of the identified *COPT* genes in *Kandelia obovata* to map the genomic chromosomal distribution of those genes to the appropriate chromosomes. All four *COPT* genes, including *KoCOPT1*, *KoCOPT2*, *KoCOPT3*, and *KoCOPT4*, were located on chromosome 1 (Chr1), chromosome 3 (Chr3), chromosome 5 (Chr5), and chromosome 10 (Chr10) (Figure 2; Table 1), respectively. 

### 2.3. Variation across the COPT Family in Terms of Domain and 3D Structure

**Figure 3:**
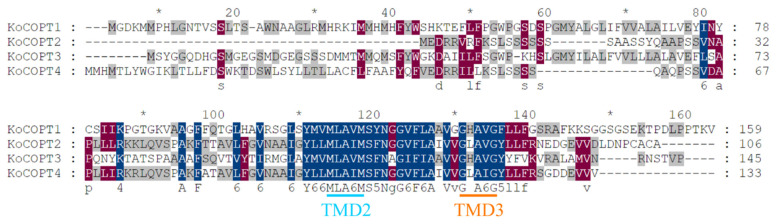
The amino acid sequence was repeatedly aligned using data from every *KoCOPT* transporter. Light blue and orange color text shows the expected transmembrane (TMD1, TMD2, TMD3) regions. Different colored bold lines indicate predicted domains (Met motifs, MxxxM, GxxxG, and CxC motifs). The dark blue color text showed 100%, dark red color text showed 80% and gray color text showed 60% sequence identity and sequence similarity. “*” above the sequence mean every ten amino acid residues.

**Figure 4:**
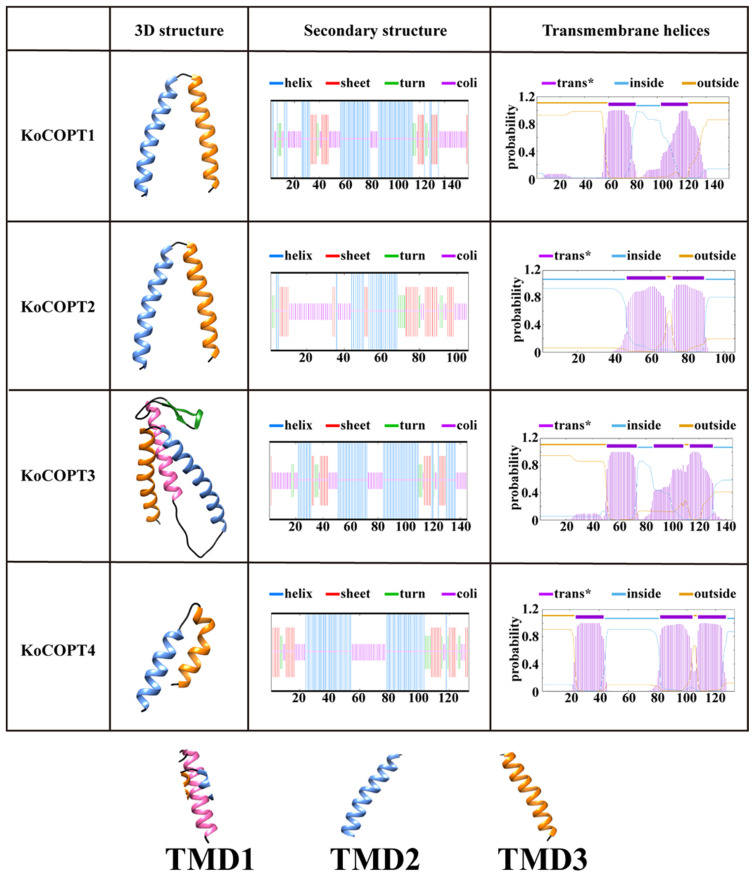
The KoCOPT 3D and transmembrane structures. The SWISS-MODEL predicts 3D structural homology models. The SOSUI tool confirmed transmembrane structures. Trans*, showed transmembrane.

BLAST in NCBI aligned all KoCOPTs, which share 60 to 80% amino acid sequence identity (Figure 3). According to a domain analysis (PF04145) of *Kandelia obovata,* COPTs (KoCOPT1, KoCOPT2, KoCOPT3, and KoCOPT4) were found in three transmembrane (TMD1, TMD2, and TMD3) domains (Figure 3 and Figure 4). The SWISS-MODEL workspace and SOSUI tool confirmed the KoCOPTs protein structures (Figure 4). TMD2 and TMD3 could be essential for Cu transport since they were highly conserved in all analyzed species. The *Kandelia obovata* COPT proteins feature secondary structures (Figure 4) comparable to COPT proteins from other species and share 60% to 80% sequence identity and sequence similarity.

### 2.4. COPT Protein Phylogenetic Relationships

**Figure 5:**
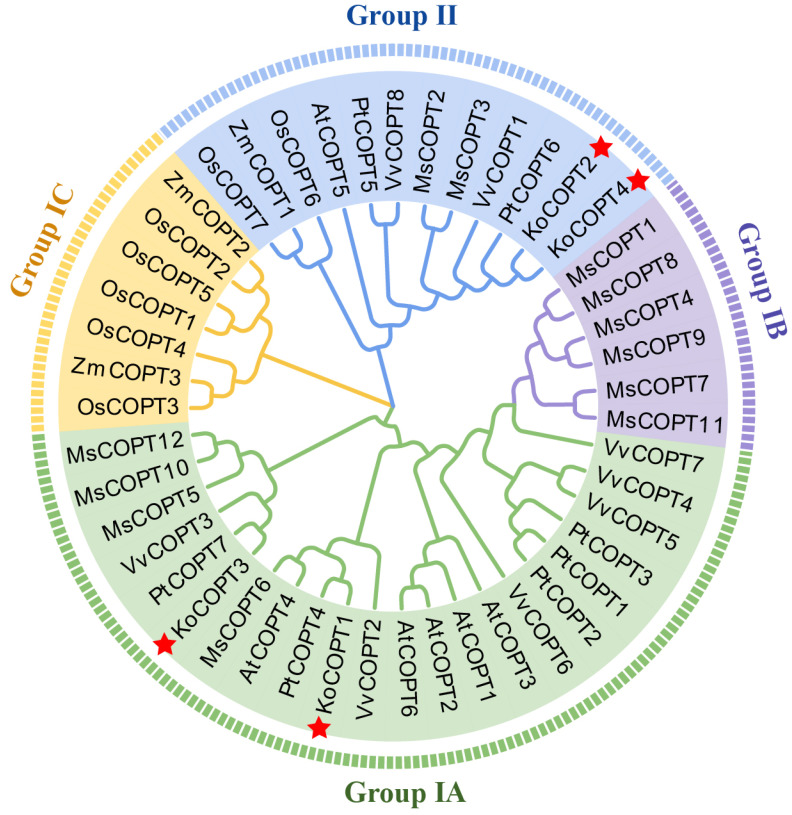
A phylogenetic analysis of COPT proteins from *Kandelia obovata* (Ko), *Arabidopsis thaliana* (At), *Oryza sativa* (Os), *Medicago sativa* (Ms), *Zea mays* (Zm), *Populus trichocarpa* (Pt), and *Vitis vinifera* (Vv) was carried out using the maximum likelihood method. There are two groups (Group I and II) of COPT proteins: Group IA, Group IB, Group IC, and Group II, each represented by a different color. Red color star “*” Showed *Kandelia obovata* NRAMP proteins.

To characterize the phylogenetic relationships among COPT proteins from *Kandelia obovata* (Ko), *Arabidopsis thaliana* (At), *Oryza sativa* (Os), *Medicago sativa* (Ms), *Zea mays* (Zm), *Populus trichocarpa* (Pt), and *Vitis vinifera* (Vv), an unrooted NJ tree was constructed aligning four KoCOPT, six AtCOPT, seven OsCOPT, twelve MsCOPT, three ZmCOPT, seven PtCOPT, and eight VvCOPT. According to the phylogenetic tree, COPT proteins could be classified into two groups. Group IA included two KoCOPT proteins (KoCOPT1/3), five AtCOPT proteins (AtCOPT1/2/3/4/6), four MsCOPT proteins (MsCOPT5/6/10/12), five PtCOPT proteins (PtCOPT1/2/3/4/7), and six VvCOPT proteins (VvCOPT2/3/4/5/6/7). Group IB included six MsCOPT proteins (MsCOPT1/4/7/8/9/11), and Group IC included five OsCOPT proteins (OsCOPT1/2/3/4/5) and two ZmCOPT proteins (ZmCOPT2/3). Group II included two KoCOPT proteins (KoCOPT2/4), one AtCOPT protein (AtCOPT5), two MsCOPT proteins (MsCOPT2/3), two PtCOPT proteins (PtCOPT5/6), two VvCOPT protein (VvCOPT1/8), one ZmCOPT protein (PtCOPT1), and two OsCOPT proteins (OsCOPT6/7). Therefore, Group I (A, B, C) had more COPT members than Group II (Figure 5).

### 2.5. COPT Gene Structure and Conserved Motif Investigation

**Figure 6:**
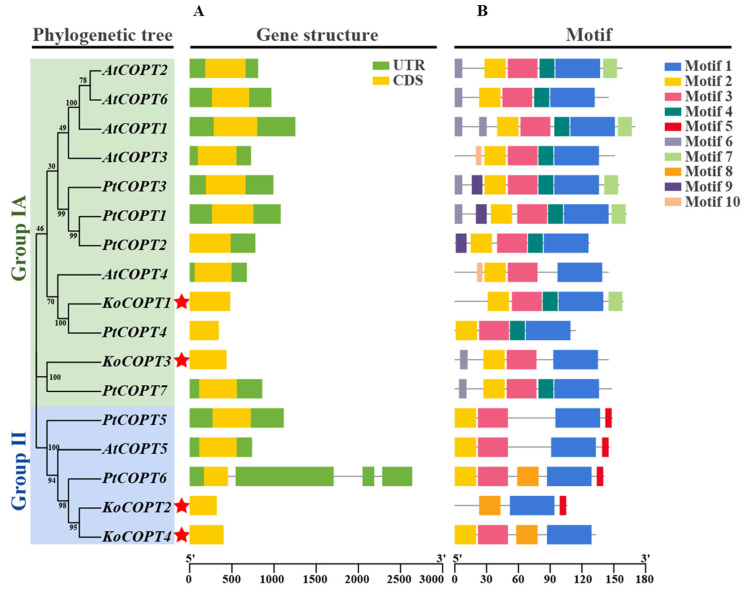
The *COPT* family genes from *Kandelia obovata* were analyzed for gene structure and motif composition. Both genomes’ *COPT* genes were grouped into two categories based on their evolutionary relationships. (**A**) The *COPT*s’ gene structure. The green color box shows the UTR regions, the yellow color box shows the CDS or exons, the black horizontal line shows the introns. (**B**) Conserved domain structures identified in the *COPTs*. Different color boxes show different motifs. Red color star “*” Showed *Kandelia obovata* NRAMP proteins.

A phylogenetic tree was made using each sequence of the COPT protein. The COPT proteins were classified into two groups, and this tree corresponded to the phylogenetic groups detailed later. The homologous gene pairs with high sequence identities displayed close evolutionary links and comparable exon–intron arrangements, as shown in Figure 6A. To further our comprehension of the evolution of the *COPT* family of genes, the exon-intron configurations of the *COPT* genes were studied. One COPT protein exon was discovered (Figure 6B). In contrast to Group II, which had the most introns (three), *PtCOPT6* only had three introns.

Similarly, each gene in Groups IA and II only had one exon. The findings indicated that group members had comparable gene structures commensurate with their evolutionary relationships because Groups I and II showed similar exon phylogenetic patterns. The protein sequences of COPTs were analyzed, and 10 conserved motifs were obtained (Figure 6B). The conserved motifs of all *COPT* genes ranged from three to seven, while the *KoCOPT* genes motifs ranged from three to five. Due to the prediction, most COPT proteins have motifs one, two, three, and four. Motif 1 was recognized in all genes, while motifs 2 and 3 were recognized in 16 genes except *KoCOPT2*. Motif 4 was identified in ten genes, while motifs 5, 6, 8, 9, and 10 were recognized in four, seven, five, three, three, and two genes, respectively.

### 2.6. Identification of Cis-Regulatory Elements in the Promoters of Four COPT Genes

**Figure 7:**
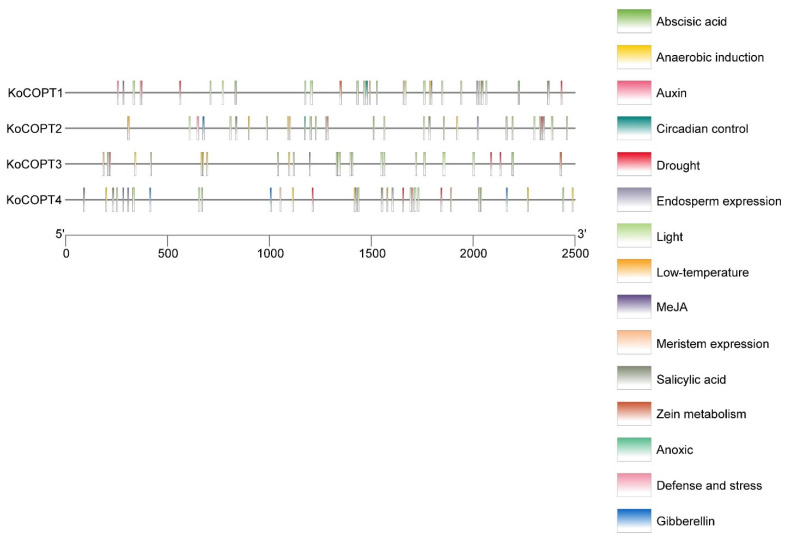
Regulatory elements known as CREs can be discovered in the *KoCOPT* gene promoters. Vertical bars display the positional distribution of the projected CREs on the *KoCOPT* promoters. PlantCARE was used to analyze the promoter sequences (2500 bp) of four *KoCOPT* genes. In this legend, each color represented different cis-elements.

After examining the 2500 bp upstream promoter sequences of *COPT* genes, we looked at the cis-element research, which may offer insights into regulatory gene expression pathways. Concerning the *COPT* genes, the light-responsiveness gene has the highest number of cis-elements (70). In addition, 45 cis-elements were involved in phytohormone (i.e., abscisic acid (15), gibberellin (4), auxin (5), salicylic acid (7), and MeJA (14)). One defense and stress, low temperature (4), drought (8), meristem expression (8), endosperm expression (3), zein metabolism (2), anoxic (1), and circadian control (1) responses were also identified in the promoter sequences of *COPT* genes (Figure 7). The variation in the response components demonstrated the regulatory functions of *COPT* genes in numerous physiological and biological processes.

### 2.7. Synteny and Duplication Analysis of the COPT Family

**Figure 8:**
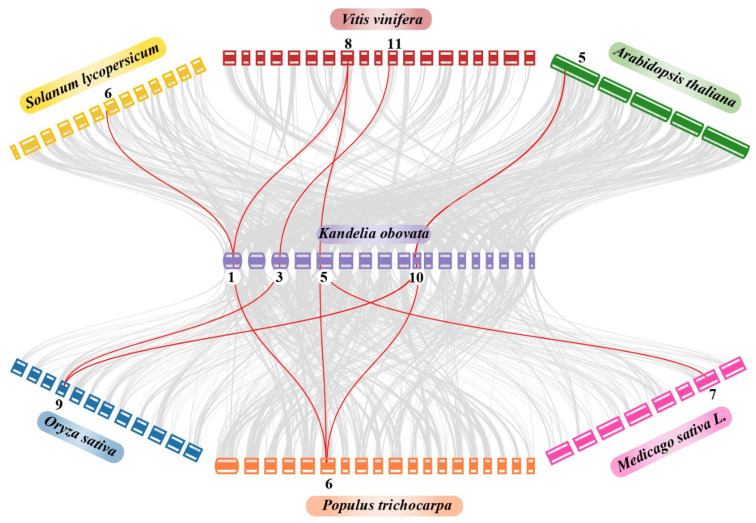
Synteny analysis of *COPT* genes in *Kandelia obovata*, *Arabidopsis thaliana*, *Oryza sativa*, *Medicago sativa*, *Solanum lycopersicum*, *Populus trichocarpa*, and *Vitis vinifera* chromosomes. The background’s grey lines emphasize the syntenic *COPT* gene pairs, while the red lines highlight the collinear blocks in the genomes of *Kandelia obovata* and the other six plant species. The box’s different colors represented various plant species. The chromosome number of each species is labeled at the top and bottom of each chromosome.

According to a collinearity analysis, *Kandelia obovata* and the other six inherited plant species, including *Arabidopsis thaliana*, *Oryza sativa*, *Medicago sativa*, *Solanum lycopersicum*, *Populus trichocarpa*, and *Vitis vinifera,* all have substantial orthologs of the *COPT* genes (Figure 8). Briefly, in chromosome 1, one *Kandelia obovata* gene displayed syntenic associations with one *Solanum lycopersicum*, *Populus trichocarpa*, and *Vitis vinifera* gene. On the other hand, in chromosome 3, one *Kandelia obovata* gene shows syntenic associations with one *Oryza sativa* and one *Vitis vinifera* gene. Similarly, in chromosome 5, one *Kandelia obovata* gene displayed syntenic associations with one *Medicago sativa, Populus trichocarpa*, and *Vitis vinifera* gene. In chromosome 10, one *Kandelia obovata* gene shows syntenic associations with one *Arabidopsis thaliana, Oryza sativa*, and *Populus trichocarpa* gene (Figure 8). Notably, several homologs of *Kandelia obovata (KoCOPTs)* survived a syntenic association with *Arabidopsis thaliana*, *Oryza sativa*, *Medicago sativa*, *Solanum lycopersicum*, *Populus trichocarpa*, and *Vitis vinifera*, indicating that segmental repetition and whole-genome duplication were important factors in the evolution of the *KoCOPT* gene family.

**Figure 9:**
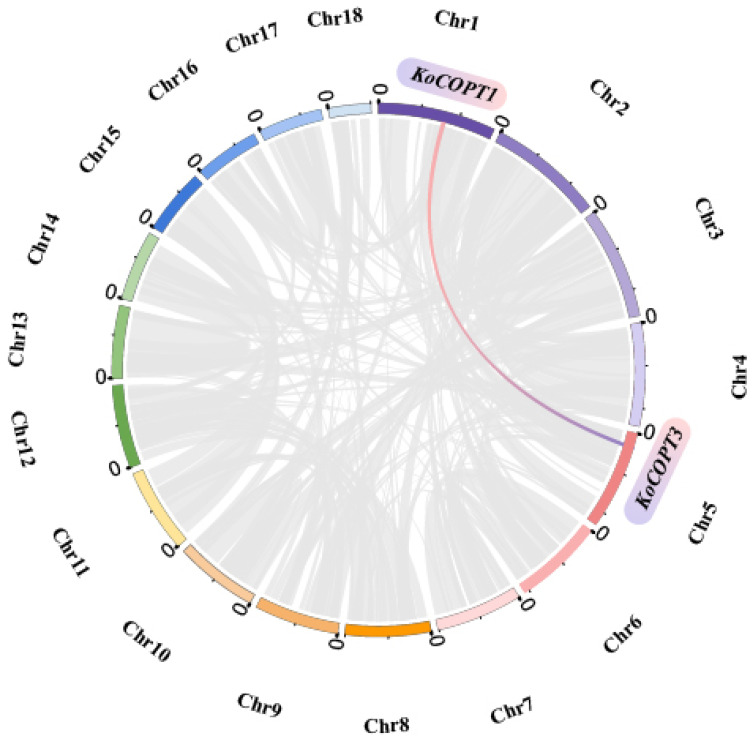
*KoCOPT* gene chromosomal dispersal and inter-chromosomal interactions are depicted in circles. The red and blue lines represent the syntenic *COPT* gene pair, while the grey lines in the background depict the syntenic blocks in the *Kandelia obovata* genome.

Segmental and tandem duplication encourages the evolution of new gene families and plant genomes. To further understand the *Kandelia obovata COPT* gene duplication activities, the segmental and tandem duplication actions in the *KoCOPT* gene family were investigated. Four *KoCOPT* genes’ chromosomal dispersals were assessed. Figure 9 demonstrates that only one tandem duplication of the *KoCOPT1* and *KoCOPT3* gene pairs of chromosomes A01 and A05 was found. Notably, the *KoCOPT* gene was absent from the remaining chromosomes. No tandem repeat paralogous genes were discovered in regions A01, A03, A05, and A10, which all have a single gene regardless of the chromosome. These findings demonstrated the significance of the duplication actions in expanding the *KoCOPT* family of genes.

To better comprehend the evolutionary limitations of the *KoCOPT* gene family, measurements of the Ka, Ks, and Ka/Ks ratio of *Kandelia obovata* were made. The duplicated *KoCOPT* gene pairs exhibited a Ka/Ks ratio of 1, which suggests that the *COPT* family genes in *Kandelia obovata* may have been subject to selection pressure or a discriminating load throughout their evolutionary history (Table 2).

### 2.8. Expression Analysis of KoCOPT Genes under Different Copper Stress

**Figure 10:**
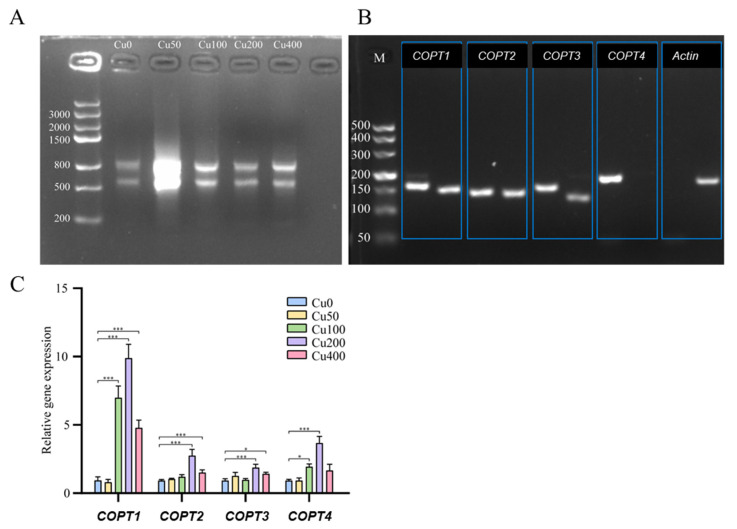
RNA electrophoresis, *COPT1-4* PCR amplification analysis, and RT-PCR measurement of the relative expression levels. (**A**) Electrophoresis of RNA extract from the leaves of *Kandelia obovata* seedling; (**B**) electrophoresis of PCR-amplified *COPT1–4* in cDNA; M represents the DNA marker. We designed two pairs of primers for each COPT gene amplification and showed two bands. For actin, we used just one primer. (**C**) qRT-PCR analysis of the expression of *KoCOPTs* in *Kandelia obovata* seedling-stage leaves under various Cu stress conditions (Cu0, Cu50, Cu100, Cu200, and Cu400 mg/L). A significant difference (*p* < 0.05) exists between the control and all conditions, according to the least significant difference (LSD) test. * = Displayed significant differences, * *p* < 0.05, *** means *p* < 0.001. The vertical axis shows the relative gene expression, and the horizontal axis shows *COPT1*–*4* genes.

Leaves at the seedling stage of *Kandelia obovata* were obtained following two years of exposure to different concentrations of copper (Cu0, Cu50, Cu100, Cu200, and Cu400 mg/L). Subsequently, total RNA was effectively extracted and subjected to reverse transcription to generate complementary DNA (cDNA) (Figure 10A). The PCR amplification of this cDNA was performed using two specific primers for each gene and showed two bands, except *COPT4*. For actin, we used just one primer and showed one band in Figure 10B. Subsequent agarose gel electrophoresis analysis confirmed the presence of two bands of the expected size. The control cDNA (Cu0) was utilized for gene amplification, as depicted in Figure 10B. In order to examine the expression patterns of four *KoCOPT* (*COPT1*, *COPT2*, *COPT3*, and *COPT4*) genes under five distinct Cu stress conditions (Cu0, Cu50, Cu100, Cu200, and Cu400 mg/L), qRT-PCR-based expression profiling was used. When Cu was limited (Cu200), *KoCOPT* expression levels increased in the leaf; however, when Cu was abundant (Cu400), these genes’ transcript levels reduced compared to the control (Cu0) and other Cu levels. Compared to the control (Cu0), all four genes showed an up-regulation with higher and more significant values with Cu-varied doses (Cu200 and Cu400). Using qRT-PCR demonstrated that two genes’ expression levels—*COPT1* and *COPT4*—were up-regulated in Cu100 but down-regulated and non-significant in Cu50 (Figure 10C and Figure S1). Compared to the control (Cu0), the expression of all four *COPT* genes in Cu50 was negligible. These *KoCOPT* genes may function as either positive or negative regulators in response to Cu stress, according to the results of qRT-PCR.

## 3. Discussion

Copper (Cu) is an essential micronutrient for living organisms. The uptake of Cu across the cell membrane is a crucial stage in regulating Cu homeostasis. There have been reports of several transporter protein variations that can mediate Cu absorption [1]. However, nothing is known about how the *COPT* genes in the typical woody mangrove *Kandelia obovata* react to Cu stress. Using genome-wide analysis in the current study, we identified a contracted *COPT* gene family with four members from the *Kandelia obovata* genome. This is fewer than the number of *COPT* genes found in other plant species, including six in *Arabidopsis thaliana* [36], seven in *Oryza sativa* [1], twelve in *Medicago sativa* [37], three in *Zea mays* [38], seven in *Populus trichocarpa* [6], and eight in *Vitis vinifera* [18]. According to published studies, several plant species have three to twelve *COPT* genes, and our result is consistent with a previous finding.

In our study, we found that different Cu treatments significantly affected the leaf width and length. These findings are in accordance with earlier research by Minwei Chai et al. [39], who predicted that under multiple heavy metal stresses, *Kandelia obovata* would expend more energy on leaf growth. In the current study, there were no appreciable differences in the height of the *Kandelia obovata* plants between the four treatments (Cu50, Cu100, Cu200, and Cu400 mg/L) and the heavy-metal-free control (Cu0). These findings support earlier research by Cheng et al. [32], who found that multiple heavy metal stresses during the 120-day trial had no appreciable impact on plant growth.

Understanding *KoCOPT* subcellular localization is critical for determining its function. All *KoCOPTs* have been shown to localize to the plasma membrane in *Kandelia obovate*, and this observation was consistent with the previous study in *A. thaliana*, which is similar to *AtCOPT1*, *AtCOPT2*, and *AtCOPT6* proteins [16]. *KoCOPTs* and Cu transporter homologs from *Arabidopsis thaliana* [28], *Oryza sativa* [1], *Medicago sativa* [37], *Zea mays* [38], and *Populus trichocarpa* [6] share a conserved structure. As an illustration, each *KoCOPT* has three TMs, and the conserved residues were primarily localized to the three TMs, providing evidence of functional conservation of these *KoCOPTs*. According to Beaudoin et al. [8], TMD2 and TMD3 are expected to be crucial in the uptake of Cu and the assembly of *PtCOPTs* as a heterotrimeric complex. It is interesting to note that *PtCOPT4* only possessed the TMD1 and TMD2 motifs, suggesting that compared to other *PtCOPTs*, a particular function may have developed, which is consistent with our results. According to [6], *PtCOPT5* and *PtCOPT6* share a CxC pattern that helps them deal with high Cu concentrations. Using the SOSUI tool and SWISS-MODEL workspace, the PtCOPT protein structures were verified. Only one TMD (*PtCOPT7*) was identified in all PtCOPT proteins as a secondary TMD, necessitating other TMD interactions to enable transport across the cell membrane [40]. Cu transport within the membrane and absorption both depend on the MxxxM motif. TMD2 deficiency may impair Cu transport or make organisms toxic [37,38]. The amino-terminal Met-rich motif in all three *ZmCOPTs* can sequester Cu (I) to the Cu transport channel; however, the quantity of this motif varies [38]. Remarkably, our outcomes aligned with the previously published findings, and the regulation and functionality of *COPT* genes may be impacted by these yet-to-be unidentified motifs.

The gene structure of *KoCOPTs* was analyzed and revealed only one nine exon; similarly, all genes in Groups IA and II contained only one exon, and *PtCOPT6* contained only three introns. The previous gene structure pattern matches *Populus trichocarpa* and *Arabidopsis thaliana*. The number of exons and introns increased due to changes in evolutionary processes brought about by changes in gene structure. Genes with fewer introns appear to undergo editing and exit the nucleus sooner in general [41,42]. This suggests that the expression pattern of *KoCOPT* genes may be influenced by variations in gene structures. Plants’ *KoCOPT* family has undergone very little evolutionary modification. The phylogenetic tree revealed that *COPT* genes from *Kandelia obovata* and six other plant species, including *Arabidopsis thaliana* [36], *Oryza sativa* [1], *Medicago sativa* [37], *Zea mays* [38], *Vitis vinifera* [18], and *Populus trichocarpa* [6], were classified into two main groups. These results suggest a possible evolutionary pattern within the *KoCOPT* gene family. All of the COPT proteins for *Kandelia obovata* were split between the two groups in accordance with the earlier classifications. Tandemly duplicated genes are more likely to be preserved during evolution when they are engaged in responses to environmental stimuli [6,43].

Promoter sequences containing cis-regulatory motifs and elements are used to better understand how the *KoCOPT* genes respond to various environmental situations. Among them were those that responded to abscisic acid, gibberellin, auxin, salicylic acid, MeJA, defense and stress, low temperature, drought, meristem expression, endosperm expression, zein metabolism, anoxic, circadian control, and other extreme cis-elements. Previous research has found that cis-elements increase plant stress responses. Our result is consistent with a previous finding: promoter cis-elements reportedly greatly influence the transcriptional control of genes in plants during tissue- or stress-specific expression patterns [6]. Given that it was discovered in the promoters of the high-affinity Cu transporters *AtCOPT1* and *AtCOPT2* in *A. thaliana*, the GTAC motif is likely implicated in Cu regulation [14,16].

According to numerous studies, *COPTs* are known to play a part in several heavy metal stress reactions. This investigation focused on how Cu stress affected *KoCOPT* expression. The expression of five rice *COPTs* was likewise impacted by increased Cu levels [1]. *PtCOPT* expression increased when Cu was limited; conversely, these genes’ transcript levels fell when Cu was abundant [6]. The *AtCOPT1* and *AtCOPT2* genes in *A. thaliana*, essential for Cu absorption under Cu deprivation, exhibit comparable expression patterns [28]. *MsCOPT* genes had significant leaf expression levels in *Medicago sativa*, which confirmed our findings with those of Shen et al. [29]. Additionally, by examining their distinct pattern of expression in leaves, we were able to show that Cu affects the expression of particular Cu transporters in *Kandelia obovata*, which is consistent with a role in Cu transport (identification of a copper transporter family in arabidopsis thaliana). *KoCOPT* genes may also be useful genetic modifiers for crops and plants to tolerate high Cu levels.

## 4. Materials and Methods

### 4.1. Identification and Characterization of COPT Genes in Kandelia obovata

The genome sequences for *Kandelia obovata* were retrieved using the NCBI database (https://www.ncbi.nlm.nih.gov/ accessed on 8 April 2023. BioProject/GWH, accession codes: PRJCA002330/GWHACBH00000000), as well as the *Kandelia obovata* protein database (https://www.omicsclass.com/article/310, accessed on 8 April 2023), were also used [22]. We used two databases to verify hypothetical proteins’ NCBI CDD: https://www.omicsclass.com/article/310 (accessed on 8 April 2023) (E-value 1.2 × 10^−28^) and Pfam: http://pfam.xfam.org/ (accessed on 8 April 2023). A protein sequence analysis of COPT associated with the domain profile was performed using the Pfam database (http://pfam.xfam.org, retrieved on 8 April 2023). The *Kandelia obovata* genome database (https://www.omicsclass.com/article/310) and NCBI database (https://www.ncbi.nlm.nih.gov/) were used to identify and confirm four *COPT* family genes (*KoCOPT1*, *KoCOPT2*, *KoCOPT3*, and *KoCOPT4*) (accessed on 8 April 2023). We used Protparam (http://web.expasy.org/protparam/) for physicochemical properties, accessed on 8 April 2023.

### 4.2. Chromosomal Distribution of COPT Genes in Kandelia obovata

The genomic positions and protein sequences of all the *COPT* genes in *Kandelia obovata* were determined using https://www.omicsclass.com/article/310 and the NCBI database, accessed on 8 April 2023. We also assessed the distribution positions of *COPT* genes on chromosomes. With the use of MapGene2Chromosome (MG2C; accessed on 8 April 2023 at http://mg2c.iask.in/mg2cv2.0/), *COPT* genes were located on the chromosomes of *Kandelia obovata*.

### 4.3. Phylogenetic Tree Construction

The phylogenetic analysis was performed using the protein sequences of *COPT* genes from the following species: *Kandelia obovata* (Ko), *Arabidopsis thaliana* (At), *Oryza sativa* (Os), *Medicago sativa* (Ms), *Zea mays* (Zm), *Populus trichocarpa* (Pt), and *Vitis vinifera* (Vv). MEGA11 (V 6.06) (www.megasoftware.net, accessed on 8 April 2023) was frequently used to align the protein sequences. The neighbor-joining (NJ) method with 1000 bootstrap repeats was used to build the phylogenetic tree. On 8 April 2023, the phylogenetic tree was viewed and edited using Fig Tree V1.4.4 [44,45].

### 4.4. Gene Structure and Significant Motif Analyses of the COPT Family Members

Four genes from the *COPT* family have been found in the *Kandelia obovata* genome. Web software (http://gsds.cbi.pku.edu.cn, accessed on 8 April 2023) determined four *COPT* genes’ structural analyses, which also displayed the exon/intron arrangements of the *COPT* genes. The online tool MEME v5.4.1, available at https://meme-suite.org/meme/tools/glam2scan, viewed on 8 April 2023, revealed more conserved strings or regions among the protein sequences of the four COPT proteins. Sequence alphabet DNA, RNA, or protein; site distribution zero or one occurrence per sequence (zoops); motif finding mode classic mode; and ten motifs were the settings utilized by the application. After downloading the related mast file, the MEME results were shown using the TBtools application [44,45].

### 4.5. Analysis of the COPT Family’s Promoter Sequences in Kandelia obovata

Utilizing the *Kandelia obovata* genome assembly database, two thousand five hundred (2500bp) upstream sequences of *COPT* family members were collected. PlantCARE (http://bioinformatics.psb.ugent.be/webtools/plantcare/html/ accessed on 8 April 2023) was used to identify CREs from the retrieved sequences. On 8 April 2023, I was able to obtain some information. The most common CREs were used to create Figure 7 in TBtools for the *COPT* genes after the frequency of each CRE motif was counted.

### 4.6. Synteny and Duplication Analysis

Synteny relationships of *COPT* genes were developed by Minspan (available online: accessed on 08 April 2023) from *Kandelia obovata* (Ko), *Arabidopsis thaliana* (At), *Oryza sativa* (Os), *Medicago sativa* (Ms), *Zea mays* (Zm), *Populus trichocarpa* (Pt), and *Vitis vinifera* (Vv). To analyze the evolutionary constraints of each *COPT* gene pair, the KaKs Calculator 2.0 (https://sourceforge.net/projects/kakscalculator2/, accessed on 8 April 2023) was used to calculate the synonymous (Ks), non-synonymous (Ka), and Ka/Ks ratios. 

### 4.7. Three-Dimensional Structure and Subcellular Localization

Use the SWISS-MODEL (https://swissmodel.expasy.org/interactive, accessed on 8 April 2023) to estimate the three-dimensional (3D) structure. Two online tools were used to predict the subcellular localization of the *COPT* family genes.

(1) ProtComp 9.0: http://linux1.softberry.com/berry.phtml?topic=protcomppl&group=programs&subgroup=proloc (accessed on 8 April 2023);
(2) CELLO server: cello.life.nctu.edu.tw/, accessed on 8 April 2023).

### 4.8. Plant Material and Environmental Conditions

One-year-old Kandelia obovata seedlings were used in the studies and were planted in Golden Bay Mangrove Reserve in Beihai, Guangxi Province, in the mangrove conservation site ((109.22° N, 21.42° E). Semi-natural cultivation methods included ensuring enough light, irrigating the soil with copper (CuCl_2_) irrigation every six months, and watering it with the local seawater every morning and evening. For two years, five different copper (CuCl_2_) concentrations (0, 50, 100, 200, and 400 mg/L) were used as the Cu0, Cu50, Cu100, Cu200, and Cu400 treatments, respectively. The control treatment, which solely included local seawater, employed a Cu0 mg/L concentration as the starting point. Leaf samples were collected to assess the parameters after two years of treatment.

### 4.9. Quantitative Real-Time PCR Assays

Total RNA was extracted from the leaves mentioned above using TRIzol (Invitrogen, http://www.invitrogen.com; accessed 8 April 2023). ABI PRISM 7500 Real-time PCR Systems (Applied Biosystems, Waltham, MA, USA) using the 2^−∆∆CT^ technique were used to carry out quantitative real-time PCR (qRT-PCR) tests, as previously described by Sun et al. [21]. Using the sequence provided by Sun et al. [21], the actin gene of *Kandelia obovata* (KoActin) was utilized as a reference gene (forward primer: CAATGCAGCAGTTGAAGGAA, reverse primer: CTGCTGGAAGGAACCAAGAG). Table 3 lists the specific *KoCOPT* gene primers used for real-time PCR, which were designed using the PRIMER 5.0 program (http://www.premierbiosoft.com/ accessed 8 April 2023) [45]. The specificity of the amplifications was verified through the observation of a single melting curve peak and the presence of a single band in agarose gel electrophoresis. For gene amplification, we used the control RNA (Cu0). For the PCR procedure, we followed our recently published article [46].

### 4.10. Statistical Analysis

Statistix 8.1 (Analytical Software, Tallahassee, FL, USA) used one-way ANOVA to analyze the data, and the findings were displayed as the mean SD (Standard Deviation) of the three replicates. Using an LSD (least significant difference) test at *p* < 0.05, the differences in the leaf mean values among five different Cu stress (Cu0, Cu50, Cu100, Cu200, and Cu400 mg/L) plants were investigated [47]. The graphs were created using the GraphPad Prism version 9.0.0 statistical program for Windows, GraphPad Software, San Diego, California USA (https://www.graphpad.com; viewed on 8 April 2023) [46].

## 5. Conclusions

**Figure 11:**
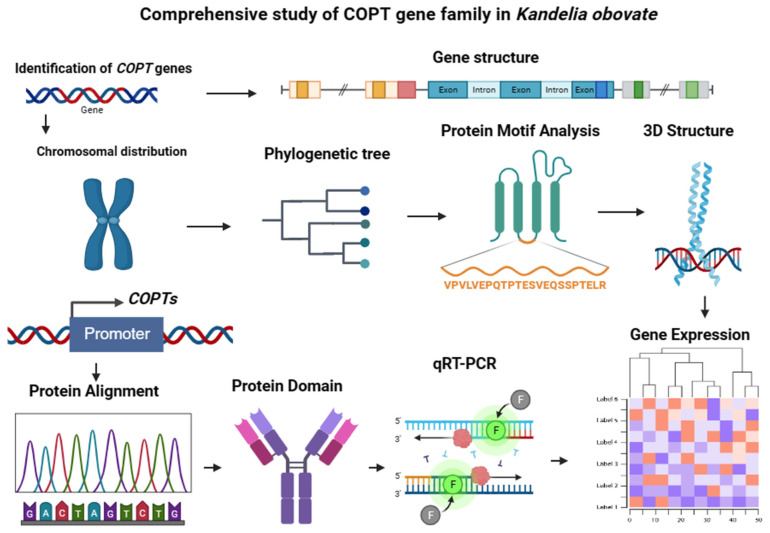
An overview of the thorough investigation of the *COPT* gene family in *Kandelia obovata*. The BioRender (https://www.biorender.com/ (accessed on 20 September 2023)) tool was used to create the figure (modified from Sajjad et al. [48]). Sketches from various analyses were displayed in different colors. The arrow sign denotes the many analyses carried out to gain an understanding of the *KoCOPT* genes characterization.

Finally, four *KoCOPT* genes were discovered in the *Kandelia obovata* genome. Domain and 3D structural variation, gene structure, phylogenetic and synteny, chromosomal distributions, motif analysis, subcellular localization, cis-regulatory elements, and expression profiling against Cu stress treatments were all performed to gain a better understanding of the evolution of the *COPT* gene family in the *Kandelia obovata* genome (Figure 11). These *KoCOPT* genes were found on *Kandelia obovata*’s four chromosomes. *Kandelia obovata*, *Arabidopsis thaliana*, *Oryza sativa*, *Medicago sativa*, *Zea mays*, *Populus trichocarpa*, and *Vitis vinifera COPT* genes were divided into two groups. The expression patterns of four *KoCOPT* genes were validated using qRT-PCR. Furthermore, various stress-related cis-acting elements were found in the promoter regions of these *KoCOPT* genes, showing that *KoCOPT* genes may be involved in various stress responses. These discoveries will also help us uncover candidate genes that improve plant architecture under stress conditions and enable possible breeding and genetic upgrades of *KoCOPT* genes for other crops, such as knockout via the CRISPR/Cas system, overexpression, etc.

## Figures and Tables

**Table 1:** Detailed information of the *COPT* gene family identified in *Kandelia obovata*.

Name	Gene ID	Location	AA ^1^	Chains ^2^	MW ^3^/kDd	pI ^4^	GRAVY ^5^	Aliphatic Index	Instability
*KoCOPT1*	geneMaker00016256	Chr1 8617182–8617661	159	+	17.09	9.72	0.309	82.2	47.68
*KoCOPT2*	geneMaker00000230	Chr3 5129069–5129389	106	-	11.18	8.71	0.498	105.85	48.23
*KoCOPT3*	geneMaker00004869	Chr5 1473358–1473795	145	-	15.64	6.82	0.566	88.83	34.84
*KoCOPT4*	geneMaker00015010	Chr10 3212193–3212594	133	-	14.78	8.82	0.805	124.66	49.66

AA ^1^: number of amino acids; chains ^2^: positive or negative chains; MW ^3^: molecular weight; pI ^4^: isoelectric point; GRAVY ^5^: grand average of hydropathicity; Chr: chromosome.

**Table 2:** Detailed information on the Ka, Ks, and Ka/Ks ratio in *Kandelia obovata*.

Name	Method	*Ka*	*Ks*	*Ka*/*Ks*	Divergence-Time (MYA)	Duplicated type
*KoCOPT1* & *KoCOP3*	MS	1.06	0.86	1.23	28.65	Segmental

MYA: million years ago.

**Table 3:** Information about the primers used in this study’s gene expression analysis by qRT-PCR.

Gene Name	Primer Name	Sequence (5′ to 3′)
*KoCOPT1*	Forward	AAGATGATGCCACACCTCGG
	Reverse	CAACCCGGGAAGAGGAACTC
*KoCOPT2*	Forward	AACGCCGCAATCGGTTATTT
	Reverse	CAAATCGACAACCTCGCCATC
*KoCOPT3*	Forward	CTTCAGCCAAGTCACCGTCT
	Reverse	AGGAACGGTGCTATTTCGGT
*KoCOPT4*	Forward	CACTTTGCTCGCCTGTTTCC
	Reverse	ACCCAATTGCCAGTCCTACG

## Data Availability

Data is contained within the article or supplementary material.

## Supplementary Materials

The supporting information can be downloaded at https://www.mdpi.com/article/10.3390/ijms242115579/s1.
