# Supplementary material for: Genome-Wide Identification and Expression Analysis of the Copper Transporter (COPT/Ctr) Gene Family in Kandelia obovata, a Typical Mangrove Plant
Source: Int J Mol Sci. 2023 Oct 25;24(21):15579. doi: 10.3390/ijms242115579 (PMC10648262; doi:10.3390/ijms242115579)
Supplement: Supplementary file 1 [file ijms-24-15579-s001.zip › ijms-2663440-supplementary.pdf]

Supplementary Materials

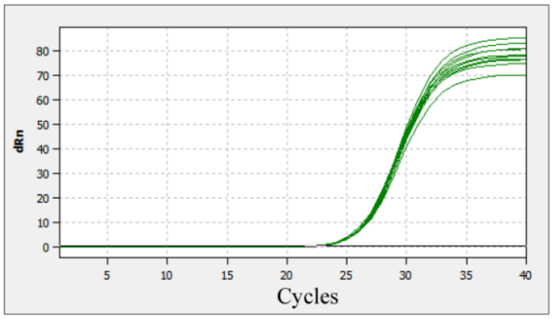

Cu0_Actin

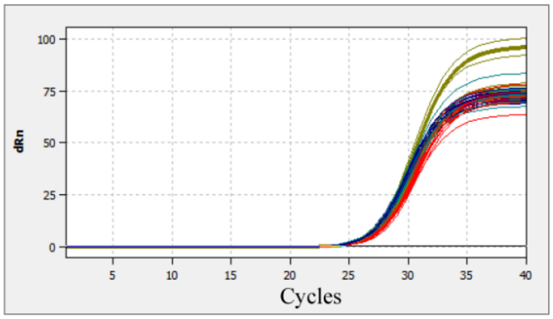

Cu50-100-200-400_Actin

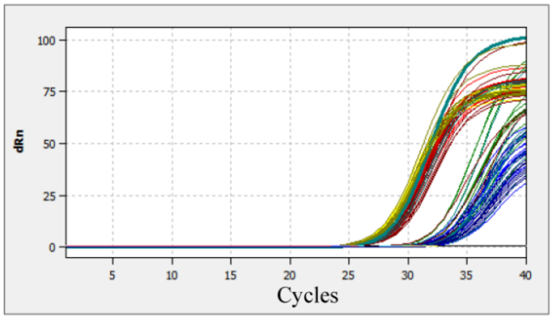

Cu0-50_COPT1-2-3-4

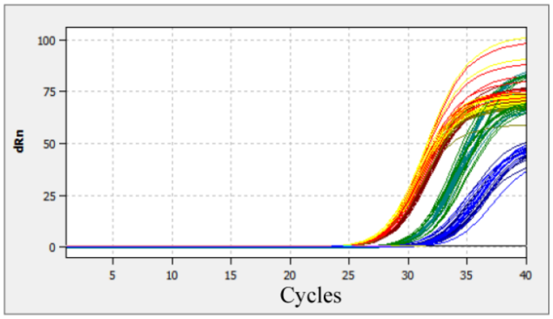

Cu100-200_COPT1-2-3-4

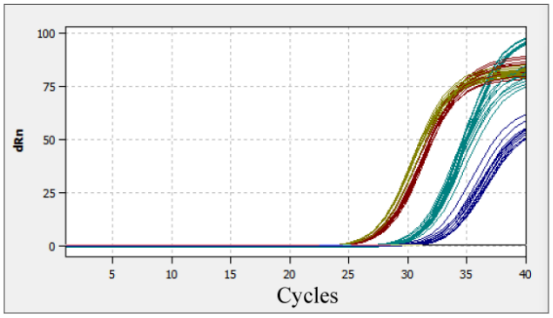

Cu400_COPT1-2-3-4

Figure S1. *COPT1-4* genes sample of curves from qPCR under five different Cu stress conditions (Cu0, Cu50, Cu100, Cu200, and Cu400 mg/L).
